# Stages of lipoedema: experiences of physical and mental health and health care

**DOI:** 10.1007/s11136-022-03216-w

**Published:** 2022-08-16

**Authors:** Chantelle Clarke, James N. Kirby, Tilly Smidt, Talitha Best

**Affiliations:** 1 NeuroHealth Lab, Appleton Institute, School of Health, Medical and Applied Sciences, Central Queensland University, Brisbane, Australia; 2 Compassionate Mind Research Group, School of Psychology, University of Queensland, Brisbane, Australia; 3 Private Practice, Heerenveen, The Netherlands

**Keywords:** Lipedema, Mental health, Physical health, Health care, Mood

## Abstract

**Purpose:**

Lipoedema is a progressive adipose (fat) disorder, and little is known about its psychological effect. This study aimed to determine the experiences of physical and mental health and health care across stages of lipoedema.

**Methods:**

Cross-sectional, secondary data from an anonymous survey (conducted 2014–2015) in Dutch and English in those with self-reported lipoedema were used (*N* = 1,362, Mdnage = 41–50 years old, 80.2% diagnosed). χ^2^ analyses of categorical data assessed lipoedema stage groups ‘Stage 1–2’ (*N* = 423), ‘Stages 3–4’ (*N* = 474) and ‘Stage Unknown’ (*N* = 406) experiences of health (physical and psychological), and health care.

**Results:**

Compared to ‘Stage 1–2’, ‘Stage 3–4’ reported more loss of mobility (*p* =  < .001), pain (*p* =  < .001), fatigue (*p* = .002), problems at work (p =  < .001) and were seeking treatment to improve physical functioning (*p* =  < .001) more frequently. ‘Stage 3–4’ were more likely to report their GP did not have knowledge of lipoedema, did not take them seriously, gave them diet and lifestyle advice, dismissed lipoedema, and treated them ‘badly’ due to overweight/lipoedema compared to ‘Stage 1–2’ (*p* =  < .001). ‘Stage 3–4’ were more likely to report depression (*p* =  < .001), emotional lability (*p* = .033) eating disorders (*p* = .018) and feeling lonelier, more fearful, and stayed at home more (*p* =  < .001) and less likely to have visited a psychologist (*p* =  < .001) compared to ‘Stage 1–2’.

**Conclusions:**

A divergent pattern of physical and psychological experiences between lipoedema stages reflects physical symptom differences and differences in psychological symptoms and health care experiences. These findings increase the understanding of lipoedema symptoms to inform psychological supports for women with lipoedema in navigating chronic health care management.

Lipoedema is a chronic health condition predominantly found in approximately 10% of women whereby symmetrical growth of fat in the hips, buttocks, thighs, and often arms occur [1]. It is described as a disease of loose connective (adipose) tissue, potentially triggered during times of hormonal change (e.g. puberty), stressful life events or surgical trauma [2]. Lipoedema progresses across three stages (higher stages reflect increasing progression) of diagnoses predominately. The criteria for stages emphasise the appearance and tactility of the increasing lipoedema tissue as opposed to the impact on joints, mobility, pain, size, or quality of life. Those with stage three lipoedema and comorbid lymphoedema have been referred to as stage four; however, lymphoedema can occur at all stages [2–5]. Current treatment methods include liposuction to remove affected tissues, diet, exercise, manual lymphatic drainage, pneumatic pumps, and compression garments to address pain, inflammation, swelling, and weight gain, as well as preserving mobility and quality of life [4, 5]. General Practitioners (GPs) play an important role in coordinating multidisciplinary teams to manage lipoedema; however, research shows significant delays in diagnosis and misdiagnosis of lipoedema as lymphoedema or general obesity and may weight shame patients [5–8]. GPs can support those with lipoedema through counselling referrals [9] and emotional support for their condition [10].

Lipoedema has a considerable negative impact on everyday functioning in terms of appearance and mobility. Often painful for individuals, the progressive abnormal distribution of fat leads to deterioration of joints and reduces mobility [3], impacting the ability to engage in physical activity, and work [6, 11, 12]. Importantly, the physical appearance of lipoedema is distressing and reduces emotional and social functioning [8]. Such that, mental health is more impaired than physical health on measures of quality of life in those with lipoedema [13]. Consequently, mental health conditions such as eating disorders, attempted suicide, depression, stress, fatigue, low self-esteem are highly prevalent in those with lipoedema [4, 6, 8, 12, 14–18].

The complex aetiology of lipoedema has contributed to the limited research that differentiates symptoms across the progression of the condition [4]. Consequently, the experiences of lipoedema and associated symptoms based on reported stages of the condition are minimal. Similarly, the experience of engagement with health care within a complex diagnostic process has yet to be explored. By understanding and exploring relevant physical, psychological and health care experiences of those with lipoedema, this study seeks to understand the clinical physical and psychological symptoms relevant to stages of lipoedema that is needed to understand, treat and support women with lipoedema.

This study aimed to determine the experiences of physical and mental health and health care across stages of lipoedema.

## Methods

### Design, setting, and sample size

This cross-sectional study is a secondary analysis of international data gathered (May 2014 to January 2015). Data were collected through purposive sampling by inviting individuals who self-identified as having lipoedema to complete the survey. Participants were recruited through online and social media via one of the largest support networks for lipoedema, based in the Netherlands. The survey was hosted in English and Dutch, and survey responses were collected anonymously at the time. The dataset was provided as a Microsoft Excel sheet and contained 1417 entries. After inspection, 11 duplicate entries were removed, leaving *N* = 1,406 entries in the dataset of which 44 entries showed missing data (*N* = 6 completed no items and *N* = 38 did not answer items relevant to the current study). Analyses reported here reflect *N* = 1,362 (*N* = 1,298 complete and *N* = 64 partially complete data entries).

### Participants

Participants were on average 41–50 years old and mainly located in the USA, *N* = 419 (29.7%) and Netherlands, *N* = 402 (28.5%), followed by the United Kingdom, *N* = 164 (11.7%), Germany, *N* = 142 (10.1%), Australia, *N* = 67 (4.8%), and Canada, *N* = 21 (1.5%), with other countries showing < 1%.

### Materials

The current study uses 51 categorical items from the dataset: demographics and comorbidities (four items: country, age, lymphoedema, and obesity), lipoedema characteristics (five items: whether participants were diagnosed, the time it took to become diagnosed, loss of mobility, pain, and fatigue), lipoedema management (seven items: sports (exercise), diet, healthy eating, stress reduction, psychological help, therapy, and liposuction) and motives for treatment (liposuction) (six items: to be thinner, more mobile, walk better, buy clothes more easily, less pain), work (four items: have a job, difficult to find a job due to lipoedema, lost a job due to lipoedema, and problems at work due to lipoedema), experiences with General Practitioners (GPs) (ten items: GP has knowledge about lipoedema, gives information about lipoedema, takes you seriously, is willing to help, is willing to learn, gives mental support, gives diet advice, gives lifestyle advice, said lipoedema is ‘bullshit’, and treated badly by GP/specialist become of lipoedema or overweight) and perceived impact on psychological characteristics and behaviours (13 items: depression, emotional lability, eating disorder, more lonely, easily depressed, quickly angry, sensitive, cry more, more fearful, inferiority complex, stay at home more, always think about lipoedema, satisfied with life), and psychological support seeking (two items: visited a psychologist about lipoedema and did it help).

### Comparison

Participants were categorised into three groups. ‘Stages 1–2’, *N* = 423 (30.0%) includes those that self-reported they were at Stage 1, *N* = 93 (6.6%), between Stage 1–2 *N* = 111 (7.9%), and Stage 2, *N* = 219 (15.5%). The second group, ‘Stages 3–4’, *N* = 474 (33.6%) includes those that reported they were between Stages 2–3, *N* = 191 (13.6%), Stage 3, *N* = 128 (9.1%), and Stage 4, *N* = 155 (11.0%). The third group, ‘Stage unknown’, *N* = 406 (28.8%) is comprised of those who reported their stage is unknown.

### Ethics

Permission to analyse and share data was obtained. Ethics approval to analyse and report the secondary dataset was provided by the Central Queensland University Human Research Ethics Committee (reference: HREC 22,882).

### Statistical analysis

Data were analysed with IBM SPSS Statistics for Windows, Version 27.0. Descriptive and non-parametric inferential statistics were used to describe the sample as a whole and to understand whether results differ significantly by stage of lipoedema. Descriptive statistics on survey items are reported for the overall sample and for each of the three groups. Kruskall–Wallis tests were used to assess group differences in age and time to diagnosis followed by Mann–Whitney *U* tests to identify differences specifically between ‘Stages 1–2’ and ‘Stages 3–4’ groups. χ^2^ analysis assessed stage group differences on the remaining items.

## Results

### Participant characteristics

Table 1 reports participants’ physical characteristics and diagnoses. The median age for the sample as a whole and for each group was 41–50 years old, with Kruskall–Wallis test identifying a significant difference between age category groups. Follow-up Mann–Whitney *U* tests showed that the Stage 1–2 group were significantly younger (*Mdn rank* = 404.8) as compared to the Stage 3–4 group (*Mdn rank* = 485.09), *z* = − 4.77, *p* < 0.001. Obesity was reported by 53% of participants and lymphoedema by 41%. All three groups differed from one another with obesity and lymphoedema most often reported by the Stage 3–4 group and least often by the Stage 1–2 group.

**Table 1:** Participant characteristics

Variable	Stage 1–2	Stage 3–4	Stage unknown	Total sample	Test statistic
Age (*Mdn*)	41–50 years	41–50 years	41–50 years	41–50 years	**χ**^**2**^**(2) = 25.46, *p* < .001**
Comorbidities (*N*, %)					
Lymphoedema					**χ**^**2**^**(4) = 94.72, *p* < .001**
Yes	111 (26.2%)^a^	273 (57.6%)^b^	155 (38.2%)^c^	539 (41.4%)	
Sometimes	29 (6.9%)^a^	23 (4.9%)^a^	31 (7.6%)^a^	83 (6.4%)	
No	283 (66.9%)^a^	178 (37.6%)^b^	220 (54.2%)^c^	681 (52.3%)	
Obesity	147 (34.8%)^a^	338 (71.3%)^b^	211 (52.0%)^c^	696 (53.4%)	**χ**^**2**^**(2) = 120.54, *p* < .001**
Diagnosis					
Diagnosed (*N*, %)	363 (85.8%)^a^	419 (88.6%)^a^	260 (64.4%)^b^	1042 (80.2%)	**χ**^**2**^**(2) = 93.03, *p* < .001**
Time to Diagnosis (*Mdn*)	11–14 years	20–25 years	20–25 years	20–25 years	**χ**^**2**^**(2) = 72.56, *p* < .001**
Physical characteristics (*N*, %)					
Loss of Mobility	76 (18.0%)^a^	249 (52.5%)^b^	105 (25.9%)^c^	430 (33.0%)	**χ**^**2**^**(2) = 134.37, *p* < .001**
Pain (pressure)	360 (85.1%)^a,b^	425 (89.7%)^b^	325 (80.0%)^a^	1110 (85.2%)	**χ**^**2**^**(2) = 16.02, *p* < .001**
Fatigue	326 (77.1%)^a^	408 (86.1%)^b^	334 (82.3%)^a,b^	1068 (82.0%)	**χ**^**2**^**(2) = 12.30, *p* = .002**
Work (*N*, %)					
Have a job	294 (69.5%)^a^	240 (50.6%)^b^	250 (61.6%)^c^	784 (60.2%)	**χ**^**2**^**(4) = 38.01, *p* < .001**
Difficult to find a job due to lipoedema	72 (17.0%)^a^	176 (37.1%)^b^	62 (15.3%)^a^	310 (23.8%)	**χ**^**2**^**(4) = 75.46, *p* < .001**
Lost a job due to lipoedema	46 (10.9%)^a^	129 (27.2%)^b^	34 (8.4%)^a^	209 (16.0%)	**χ**^**2**^**(4) = 70.26, *p* < .001**
Problems at work due to lipoedema	176 (41.6%)^a^	290 (61.2%)^b^	156 (38.4%)^a^	622 (47.7%)	**χ**^**2**^**(4) = 59.76, *p* < .001**

Bold values indicate statistically significant results (*p* < .05)

^a^, ^b^, ^c^ indicates the groups that differed significantly from one another

As shown in Table 1, those within the Stage 1–2 group and Stage 3–4 groups were significantly more likely to report receiving a formal diagnosis (86% and 89%, respectively) as compared to the Stage Unknown group in which 64% had received a formal diagnosis but did not know their stage. Median time to diagnosis was 20–25 years for all participants with follow-up Mann–Whitney *U* Test revealing that those in Stage 1–2 took less time to diagnose (*Mdn* = 11–14 years) compared to those in Stage 3–4 (*Mdn* = 20–25 years).

### Physical characteristics and impact

Participants often reported pain (85%) and fatigue (82%) and 33% reported a loss of mobility (Table 1). Post-hoc comparisons showed that a significantly higher percentage of those in the stage 3–4 group reported fatigue compared to the Stage 1–2 group alone and pain (pressure) compared to the Stage Unknown group alone. All three groups differed significantly on loss of mobility which was most often reported by the Stage 3–4 group (52%) and least reported by the Stage 1–2 group (18%).

Less than two thirds of participants had a job, with the Stage 1–2 group being the most likely to have a job, followed by the Stage Unknown Group, and then the Stage 3–4 group, with all groups differing significantly from each other (Table 1). The stage 3–4 group was also significantly more likely to report that lipoedema caused problems at work, difficulty finding a job and causing job loss as compared to both other groups.

### Treatment methods and motives

The most common method reported for managing lipoedema symptoms was healthy eating, followed by sports (exercise), dietary changes and therapies (Table 2). The least used were psychological help and liposuction. Stages 1–2 were significantly more likely to have had liposuction and use sports and less likely to use diet and psychological help as compared to both the Stages 3–4 and stage unknown groups. Those of unknown stage were significantly less likely to use therapies and healthy eating as compared to both other groups.

**Table 2:** Physical characteristics and motives for treatment

Variable	Stage 1–2	Stage 3–4	Stage unknown	Total sample	Test statistic
Management approaches (*N*, %)					
Sports (exercise)	232 (54.8%)^a^	166 (35.0%)^b^	153 (37.7%)^b^	551 (42.3%)	**χ**^**2**^**(2) = 41.11, *p* < .001**
Diet	164 (28.8%)^a^	177 (37.3%)^a^	138 (34.0%)^a^	479 (36.8%)	χ^2^(2) = 2.14, *p* = .342
Healthy eating	322 (76.1%)^a^	328 (69.2%)^a^	245 (60.3%)^b^	895 (68.7%)	**χ**^**2**^**(2) = 24.06, *p* < .001**
Stress reduction	104 (24.6%)^a^	122 (25.7%)^a^	90 (22.2%)^a^	316 (24.3%)	χ^2^(2) = 1.55, *p* = .459
Psychological help	48 (11.3%)^a^	58 (12.2%)^b^	22 (5.4%)^b^	128 (9.8%)	**χ**^**2**^**(2) = 13.11, *p* = .001**
Therapy	131 (31.0%)^a^	171 (36.1%)^a^	79 (19.5%)^b^	381 (29.2%)	**χ**^**2**^**(2) = 30.09, *p* < .001**
Liposuction (has had)	73 (17.3%)^a^	69 (14.6%)^b^	35 (8.6%)^b^	177 (13.6%)	**χ**^**2**^**(2) = 13.85, *p* < .001**
Want to have liposuction					χ^2^(4) = 7.28, *p* = .121
Yes	241 (57.0%)^a^	264 (55.7%)^a,b^	197 (48.5%)^b^	702 (53.9%)	
Perhaps	110 (26.0%)^a^	125 (26.4%)^a^	130 (32.0%)^a^	365 (28.0%)	
No	72 (17.0%)^a^	85 (17.9%)^a^	79 (19.5%)^a^	236 (18.1%)	
Liposuction motivations					
To be thinner	173 (40.9%)^a^	140 (29.5%)^b^	164 (40.4%)^a^	477 (36.6%)	**χ**^**2**^**(2) = 16.07, *p* < .001**
To be more mobile	177 (41.8%)^a^	335 (70.7%)^b^	185 (45.6%)^a^	697 (53.5%	**χ**^**2**^**(2) = 89.57, *p* < .001**
To walk better	185 (43.7%)^a^	322 (67.9%)^b^	190 (46.8%)^a^	697 (53.5%)	**X**^**2**^**(2) = 63.22, *p* < .001**
To buy clothes more easily	216 (51.1%)^a^	252 (53.2%)^a^	221 (54.4%)^a^	689 (52.9%)	χ^2^(2) = 0.96, *p* = .616
To have less pain	305 (72.1%)^a,b^	366 (77.2%)^b^	270 (66.5%)^a^	941 (72.2%)	**χ**^**2**^**(2) = 12.51, *p* = .002**

Bold values indicate statistically significant results (*p* < .05)

^a^, ^b^ indicates the groups that differed significantly from one another

Over 80% of participants wanted to have liposuction or were considering it (Table 2). The Stage 3–4 group was less likely to report they wanted liposuction to be thinner and more likely to report they wanted liposuction to increase mobility and to walk better as compared to both other groups, and decrease pain as compared to the Stage unknown group.

### Experiences in health care

Table 3 shows significant differences between groups on all measures of doctor–patient experiences. Over half reported a perception and experience of GPs as being unaware of lipoedema with the Stage 3–4 group significantly more likely to report that their GP did not have knowledge about lipoedema than both other groups. Additionally, nearly 75% reported their GP did not provide information about the condition with the ‘Stage 3–4’ group significantly more likely to report not being provided with information than the ‘Stage Unknown’ group. Overall, 60% reported their GP took them seriously at least a little bit. The Stage 3–4 group was more likely to report that their GP did not take them seriously as compared to the Stage 1–2 group, and the Stage Unknown group was less likely to report that their GP did take them seriously compared to both other groups. Only 58% of participants perceived that their GPs were willing to help and 46% perceived their GPs were willing to learn (46%), at least a little bit, with the Stage Unknown group significantly less likely to report GPs as willing to help and willing to learn as compared to both other groups. Approximately 34% perceived their GP provided at least a little mental support. The Stage Unknown group was significantly less likely to be provided with mental support as compared to both other groups. Overall, less than a third reported their GP provided diet and lifestyle advice, with the Stage 3–4 group significantly more likely to receive diet and lifestyle advice compared to both other groups. Approximately 30% of participants reported that their GP dismissed lipoedema (called lipoedema ‘bullshit’), and 58% reported being treated badly by their GP due to lipoedema/overweight, with the Stage 3–4 group significantly more likely to report that their GP dismissed lipoedema and treated them badly compared to both other groups.

**Table 3:** Experiences in health care

Variable	Stage 1–2	Stage 3–4	Stage unknown	Total sample	Test statistic
GP has knowledge about lipoedema					**χ**^**2**^**(6) = 51.51, *p* < .001**
Yes	47 (11.1%)^a^	38 (8.0%)^a,b^	20 (4.9%)^b^	105 (8.1%)	
A little bit	68 (16.1%)^a^	71 (15.0%)^a^	66 (16.3%)^a^	150 (15.7%)	
No	260 (61.5%)^a^	340 (71.7%)^b^	243 (59.9%)^a^	843 (64.7%)	
I do not know	48 (11.3%)^a^	25 (5.3%)^b^	77 (19.0%)^c^	150 (11.5%)	
GP gives information about lipoedema					**χ**^**2**^**(6) = 33.91, *p* < .001**
Yes	26 (6.1%)^a^	32 (6.8%)^a^	15 (3.7%)^a^	73 (5.6%)	
A little bit	39 (9.2%)^a^	36 (7.6%)^a^	37 (9.1%)^a^	112 (8.6%)	
No	308 (72.8%)^a,b^	373 (78.7%)^b^	277 (68.2%)^a^	958 (73.5%)	
I do not know	50 (11.8%)^a^	33 (7.0%)^b^	77 (19.0%)^c^	160 (12.3%)	
GP takes you seriously					**χ**^**2**^**(6) = 46.6, *p* < .001**
Yes	191(45.2%)^a^	219 (46.2%)^a^	134 (33.0%)^b^	544 (41.7%)	
A little bit	82 (19.4%)^a^	91 (19.2%)^a^	76 (18.7%)^a^	249 (19.1%)	
No	86 (20.3%)^a^	130 (27.4%)^b^	111 (27.3%)^a,b^	327 (25.1%)	
I do not know	64 (15.1%)^a^	34 (7.2%)^b^	85 (20.9%)^a^	183 (14.0%)	
GP is willing to help					**χ**^**2**^**(6) = 46.90, *p* < .001**
Yes	166 (39.2%)^a^	198 (41.8%)^a^	118 (29.1%)^b^	482 (37.0%)	
A little bit	89 (21.0%)^a^	112 (23.6%)^a^	78 (19.2%)^a^	279 (21.4%)	
No	96 (22.7%)^a^	125 (26.4%)^a^	116 (28.6%)^a^	337 (25.9%)	
I do not know	72 (17.0%)^a^	39 (8.2%)^b^	94 (23.2%)^a^	205 (15.7%)	
GP is Willing to learn					**χ**^**2**^**(6) = 51.30, *p* < .001**
Yes	121 (28.6%)^a^	166 (35.0%)^a^	86 (21.2%)^b^	373 (28.6%)	
A little bit	81 (19.1%)^a^	90 (19.0%)^a^	53 (13.1%)^a^	224 (17.2%)	
No	106 (25.1%)^a^	137 (28.9%)^a^	123 (30.3%)^a^	366 (28.1%)	
I do not know	115 (27.2%)^a^	81 (17.1%)^b^	144 (35.5%)^c^	340 (26.1%)	
GP gives mental support					**χ**^**2**^**(6) = 51.36, *p* < .001**
Yes	97 (22.9%)^a^	110 (23.2%)^a^	60 (14.8%)^b^	267 (20.5%)	
A little bit	53 (12.5%)^a,b^	77 (16.2%)^b^	42 (10.3%)^a^	172 (13.2%)	
No	221 (52.2%)^a^	247 (52.1%)^a^	209 (51.5%)^a^	677 (52.0%)	
I do not know	52 (12.3%)^a^	40 (8.4%)^a^	95 (23.4%)^b^	187 (14.4%)	
GP gives diet advice					**χ**^**2**^**(6) = 52.54, *p* < .001**
Yes	78 (18.4%)^a^	124 (26.2%)^b^	72 (17.7%)^a^	274 (21.0%)	
A little bit	48 (11.3)^a^	55 (11.6%)^a^	41 (10.1%)^a^	144 (11.1%)	
No	246 (58.2%)^a^	268 (56.5%)^a^	208 (51.2%)^a^	722 (55.4%)	
I do not know	51 (12.1%) ^a^	27 (5.7%)^b^	85 (20.9%)^c^	163 (12.5%)	
GP gives lifestyle advice					**χ**^**2**^**(6) = 58.84, *p* < .001**
Yes	76 (18.0%)^a^	121 (25.5%)^b^	66 (16.3%)^a^	263 (20.2%)	
A little bit	47 (11.1%)^a^	53 (11.2%)^a^	46 (11.3%)^a^	146 (11.2%)	
No	251 (59.3%)^a^	274 (57.8%)^a,b^	207 (51.0%)^b^	732 (56.2%	
I do not know	49 (11.6%)^a^	26 (5.5%)^b^	87 (21.4%)^c^	162 (12.4%)	
GP/specialist said lipoedema is ‘bullshit’ (Yes)	106 (25.1%)^a^	199 (42.0%)^b^	87 (21.4%)^a^	392 (30.1%)	**χ**^**2**^**(2) = 51.44, *p* < .001**
Treated badly by a GP/specialist because of lipoedema or overweight					**χ**^**2**^**(4) = 126.47, *p* < .001**
Yes	148 (35.0%)^a^	302 (63.7%)^b^	135 (33.3%)^a^	585 (44.9%)	
Sometimes	51 (12.1%)^a^	66 (13.9%)^a^	58 (14.3%)^a^	175 (13.4%)	
No	224 (53.0%)^a^	106 (22.4%)^b^	213 (52.5%)^a^	543 (41.7%)	

Bold values indicate statistically significant results (*p* < .05)

^a^, ^b^, ^c^ indicates the groups that differed significantly from one another

### Psychological experiences

Overall, approximately 40% reported depression, 28% reported emotional lability and 16% reported eating disorders (Table 4). The stage 3–4 group were significantly more likely to report depression and eating disorders compared to both other groups, and emotional lability compared to the Stage Unknown group alone. Individuals self-reported that their experience of lipedema ‘changed their personality’ in terms of ‘sensitivity’ (75.8%), ‘always thinking about lipoedema’ (73.4%), ‘inferiority complex’ (72.8%), ‘staying at home more’ (67.7%), ‘easily depressed’ (65.4%), ‘more lonely’ (59.8%), ‘cry more’ (55.0%), ‘quickly angry’ (49.0%), and being ‘more fearful’ (47.4%) (Table 4). The ‘Stage 3–4’ group were significantly more likely to report that lipoedema changed their personality in terms of being more lonely, staying at home more, and being more fearful as compared to both other groups. Further, those in Stage 3–4 were more likely to report being more easily depressed, always thought about lipoedema, cried more and were quick to anger compared to the Stage unknown group alone. Just over a quarter of participants reported they were not satisfied with life, and this was significantly higher in Stage 3–4 compared to both other groups (Table 4). Few participants reported having seen a psychologist (22%), with Stage 3–4 significantly more likely to report having seen a psychologist compared to both other groups. Amongst those who had visited a psychologist (*N* = 287), 41.1% reported that it was helpful.

**Table 4:** Psychological experiences in lipoedema

Variable	Stage 1–2	Stage 3–4	Stage unknown	Total sample	Test statistic
Psychological experiences					
Depression	147 (34.8%)^a^	229 (48.3%)^b^	142 (35.0%)^a^	518 (39.8%)	**χ**^**2**^**(2) = 22.78, *p* < .001**
Emotional lability	111 (26.2%)^a,b^	152 (32.1%)^b^	100 (24.6%)^a^	363 (27.9%)	**χ**^**2**^**(2) = 6.83, *p* = .033**
Eating disorder	68 (16.1%)^a^	93 (19.6%)^b^	51 (12.6%)^a^	212 (16.3%)	**χ**^**2**^**(2) = 8.01, *p* = .018**
Perceived lipoedema impact					
More lonely					**χ**^**2**^**(4) = 25.97, *p* < .001**
Yes	153 (36.2%)^a^	227 (47.9%)^b^	134 (33.0%)^a^	514 (39.4%)	
A little bit	91 (21.5%)^a^	92 (19.4$)^a^	82 (20.2%)^a^	265 (20.3%)	
No	179 (42.3%)^a^	155 (32.7%)^b^	190 (46.8%)^a^	524 (40.2%)	
Easily depressed					**χ**^**2**^**(4) = 15.32, *p* = .004**
Yes	172 (40.7%)^a,b^	217 (45.8%)^b^	140 (34.5%)^a^	529 (40.6%)	
A little bit	99 (23.4%)^a^	120 (25.3%)^a^	104 (25.6%)^a^	323 (24.8%)	
No	152 (35.9%)^a,b^	137 (28.9%)^b^	162 (39.9%)^a^	451 (34.6%)	
Quickly angry					**χ**^**2**^**(4) = 25.78, *p* < .001**
Yes	108 (25.5%)^a,b^	154 (32.5%)^b^	101 (24.9%)^a^	363 (27.9%)	
A little bit	104 (24.6%)^a^	109 (23.0%)^a^	62 (15.3%)^b^	275 (21.1%)	
No	211 (49.9%)^a^	211 (44.5%)^a^	243 (5959%)^b^	665 (51.0%)	
Sensitive					χ^2^(4) = 7.10, *p* = .130
Yes	235 (55.6%) ^a^	280 (59.1%) ^a^	209 (51.5%) ^a^	724 (55.6%)	
A little bit	90 (21.3%) ^a^	92 (19.4%) ^a^	82 (20.2%) ^a^	264 (20.3%)	
No	98 (23.2%) ^a^	102 (21.5%) ^a^	115 (28.3%) ^a^	315 (24.2%)	
Cry more					**χ**^**2**^**(4) = 16.59, *p* = .002**
Yes	151 (35.7%)^a,b^	203 (42.8%)^b^	129 (31.8%)^a^	483 (37.1%)	
A little bit	78 (18.4%)^a^	88 (18.6%)^a^	67 (16.55%)^a^	233 (17.9%)	
No	194 (45.9%)^a,b^	183 (38.6%)^b^	210 (51.7%)^a^	587 (45.0%)	
More fearful					**χ**^**2**^**(4) = 18.47, *p* < .001**
Yes	125 (29.6%)^a^	184 (38.8%)^b^	109 (26.8%)^a^	418 (32.1%)	
A little bit	64 (15.1%)^a^	74 (15.6%)^a^	61 (15.0%)^a^	199 (15.3%)	
No	234 (55.3%)^a^	216 (45.6%)^b^	236 (58.1%)^a^	686 (52.6%)	
Inferiority complex					χ^2^(4) = 5.4, *p* = .242
Yes	208 (49.2%)^a^	260 (54.9%)^a^	202 (49.8%)^a^	670 (51.4%)^a^	
A little bit	89 (21.0%)^a^	99 (20.9%)^a^	81 (20.0%)^a^	269 (20.6%)^a^	
No	126 (29.8%)^a^	115 (24.3%)^a^	123 (30.3%)^a^	364 (27.9%)^a^	
Stay at home more					**χ**^**2**^**(4) = 53.94, *p* < .001**
Yes	188 (44.4%)^a^	305 (64.3%)^b^	185 (45.6%)^a^	678 (52.0%)	
A little bit	66 (15.6%)^a^	68 (14.3%)^a^	58 (14.3%)^a^	192 (14.7%)	
No	169 (40.0%)^a^	101 (21.3%)^b^	163 (40.1%)^a^	433 (33.2%)	
Always think about lipoedema					**χ**^**2**^**(4) = 15.45, *p* < .001**
Yes	187 (44.2%)^a,b^	231 (48.7%)^b^	150 (36.9%)^a^	568 (43.6%)	
A little bit	122 (28.8%)^a^	139 (29.3%)^a^	127 (31.3%)^a^	388 (29.8%)	
No	144 (27.0%)^a,b^	104 (21.9%)^b^	129 (31.8%)^a^	347 (26.6%)	
Satisfied with life					**χ**^**2**^**(4) = 30.23, *p* < .001**
Yes	169 (40.0%)^a^	129 (27.2%)^b^	158 (38.9%)^a^	465 (35.0%)	
Sometimes	161 (38.1%)^a^	176 (37.1%)^a^	148 (36.5%)^a^	485 (37.2%)	
No	93 (22.0%)^a^	169 (35.7%)^b^	100 (24.6%)^a^	362 (27.8%)	
Visited a psychologist about lipoedema					**χ**^**2**^**(6) = 26.29, *p* < .001**
Yes	89 (21.0%)^a^	133 (28.1%)^b^	65 (16.0%)^a^	287 (22.0%)	
No, but I want to	31 (7.3%)^a^	29 (6.1%)^a^	25 (6.2%)^a^	85 (6.5%)	
No	283 (66.9%)^a,b^	300 (63.3%)^b^	288 (70.9)^a^	871 (66.8%)	
Inapplicable	20 (4.7%)^a,b^	12 (2.5%)^b^	28 (6.9%)^a^	60 (4.6%)	
Did it (visiting a psychologist) help?					χ^2^(4) = 5.58, *p* = .232
Yes	38 (42.7%)^a^	61 (45.9%)^a^	19 (29.2%)^a^	118 (41.4%)	
No	46 (51.7%)^a^	62 (46.6%)^a^	40 (61.5%)^a^	148 (51.6%)	
Inapplicable	5 (5.6%)^a^	10 (7.5%)^b^	6 (9.2%)^a^	21 (7.3%)	

Bold values indicate statistically significant results (*p* < .05)

^a^, ^b^ indicates the groups that differed significantly from one another

## Discussion

The current study provides unique insight into lipoedema, and the negative impact experienced across physical and psychological functioning and health care. Those with lipoedema experience considerable difficulty in gaining timely and appropriate health care, increased pain and fatigue, and reduced mobility and ability to work, which in turn increases negative psychological, emotional, and social experiences. Importantly, a divergent pattern of experiences between those of higher and lower stages of lipoedema was identified. Those with stages 3–4 lipoedema were more likely to report physical difficulties and difficulty with work which in turn reduced use of exercise and increased motivation for medical treatment to reduce the physical burden of lipoedema than those in stages 1–2. Further, stages 3–4 were more likely to report difficulties in health care (not being taken seriously and exposure to weight stigma) and experience psychological and social dysfunction (depression, eating disorders, fearfulness, staying at home and loneliness) compared to stages 1–2. Despite these difficulties, few accessed psychological support. This differential profile of experience between lipoedema stage groups has not yet been fully understood.

### Physical characteristics and impact

The results of this study profile the differential effects of lipoedema stages on physical functioning. Those within stage 3–4 lipoedema were more likely to be older and have comorbid obesity and lymphoedema, likely related to the progression of lipoedema over time with age, the associated increase in Body Mass Index (BMI), and increased burden on the lymphatic system [3]. Like previous research, pain and fatigue were highly prevalent across stages [4, 6, 12, 14]. The current study showed that stages 3–4 lipoedema disproportionately experienced pain, fatigue, and mobility issues. This may be expected as mobility issues can result from damaged hip and knee joints and the development of orthopaedic disorders and gait alterations with lipoedema progression [3].

All stage groups reported a negative impact on work, mirroring previous research that shows between 51–73% are impacted in their career and restricted career choices [6, 12]. These findings extend this research by showing that those of stages 3–4 were significantly more likely to report experiencing difficulties at work and in finding and keeping a job due to lipoedema compared to stages 1–2. Qualitative research with stage 3–4 women with lipoedema found mobility issues are a primary concern, impacting everyday activities and work [11]. Thus, the higher prevalence of impact on work functioning within stages 3–4 in the current study may reflect the greater prevalence of mobility issues, pain and fatigue found in this group. Those within stages 3–4, therefore, may need additional physical supports to preserve mobility.

### Lipoedema management and motives

Results indicated that stages 3–4 are significantly less likely to exercise to manage lipoedema compared to stages 1–2, perhaps due to the higher prevalence of pain and mobility issues in this group. For example, UK research found that pain, health issues and lack of mobility drive reduced exercise in lipoedema [6]. This is concerning as exercise is an important component of conservative treatment to move lymphatic fluid, manage weight, reduce inflammation, and improve overall physical and mental health [2, 5, 19]. Increasing participation in exercise is, therefore, important, particularly for those of stages 3–4.

Increasing walking capability and mobility and reducing pain are important motivators behind wanting liposuction across stages, mirroring UK findings [12]. This is likely because liposuction treatments remove abnormal lipoedema tissues and improve pain, mobility, lymphatic functioning, social functioning, quality of life, career prospects, ability to exercise, and reduced need for care [2, 12, 13, 20, 21, 22]. This study further demonstrates that stages 3–4 are more likely to report being motivated to receive liposuction to walk better and improve mobility, and less likely to want liposuction to be ‘thinner’, as compared to stages 1–2. This is likely related to the greater impact of mobility and reduced working capacity found within stages 3–4. Despite this, those in stages 3–4 were less likely to receive liposuction treatment compared to stages 1–2. Navigating the health care system to receive appropriate diagnosis and treatments, however, can be difficult.

### Experiences in health care

A key finding was the difficulties patients experienced with GPs in lipoedema-related health care. Results showed limited awareness of lipoedema by GPs alongside significant delays in receiving a diagnosis (20–25 years). Similarly, the UK research in 2012 showed only 5% of those with lipoedema reported their GPs provided diagnosis [6] and survey research on a Dutch lipoedema website found an average of 18 years and 2.5 doctors to diagnose after onset [8]. More recently, Fetzer and Warrilow [12] showed a median of 26–40 years to diagnosis in the UK suggesting delays in diagnosis remains, with the current study showing that difficulties in diagnosis occur internationally.

The lack of awareness of lipoedema by GPs may explain why some reported resistance by GPs to treat lipoedema. For example, many participants reported that their GPs were unwilling to learn about lipoedema, unwilling to help and did not take them seriously and 30% reported their GP dismissed lipoedema as a valid health condition. This mirrors UK research that showed that many of those with lipoedema report that their doctors were unhelpful and dismissive in response to complaints of lipoedema symptoms and often misattributed them to obesity [6]. The current study showed that stages 3–4 are more likely to report not being taken seriously by their GP than stages 1–2, perhaps due to misattribution of lipoedema symptoms as being related to obesity.

Many participants reported experiencing being treated badly by their doctor/s due to their weight/lipoedema, consistent with research showing that lipoedema patients report having been blatantly ‘weight-shamed’ by doctors and told to reduce food intake and increase exercise [6, 7]. Our findings provide greater nuance, with results indicating that those of stages 3–4 are more likely to experience mistreatment due to their weight/lipoedema compared to stages 1–2, perhaps related to increased prevalence of comorbid obesity in this group or misdiagnosis/perception of lipoedema as obesity and general lack of awareness of lipoedema as a valid health condition. Health care providers are well-known sources of weight stigma. For example, a recent meta-analysis of 40 studies showed that health care providers are a source of both implicit and explicit weight biases [23]. These weight biases and stigmatisation can adversely affect the quality of care provided and delay appropriate diagnosis and care due to misattribution of symptoms to obesity, and lead to reduced health behaviours, avoidance of health care and adverse psychological outcomes [24, 25]. Further, a recent systematic review across 17 studies (*N* = 21,172) found that experienced/perceived weight stigma can become internalised, leading to negative outcomes such as body shame [26] which has been linked to increased self-criticism and depression [27]. Training GPs to identify lipoedema and targeting weight bias and stigmatisation are, therefore, important areas to address in lipoedema-related health care.

### Psychological experiences

There is significant psychological distress experienced by patients with lipoedema, which differed by stage. Approximately 40% reported depression and 16% reported eating disorders, reflective of research using validated scales showing a prevalence of depression within lipoedema between 31 and 59% [8, 16, 17] and eating disorders of 18% [17]. The results indicated that stages 3–4 are more likely to experience depression and eating disorders than those in stages 1–2. This adds to research showing that increased lipoedema symptom severity is associated with increasingly severe depression [16] and a higher prevalence of depression and eating disorders in those with a body mass index (BMI) of ≥ 40 compared to < 40 [17]. This study demonstrated symptoms of psychological distress in that those with lipoedema often experience sensitivity, rumination (always thinking about lipoedema), inferiority, loneliness, isolation (staying at home more), crying, anger, and fearfulness, which participants attributed to lipoedema. These findings indicate that those with lipoedema care about and focus on themselves and their lipoedema, often feeling inferior compared to others. Feelings of inferiority may be linked to shame and fearfulness of the judgements of others, leading to social avoidance and feelings of loneliness.

Interestingly, those in Stage 3–4 reported experiencing greater social impairment (fearfulness, loneliness and staying at home) as compared to those in Stage 1–2, extending research reporting social impairment in lipoedema [6, 8, 13]. The higher prevalence of social impairment in stages 3–4 may be linked to increased exposure to weight stigma. For example, qualitative research with 11 women with stage 3 lipoedema showed they were often exposed to weight stigma and discrimination that impacted their confidence and self-worth but led to impaired social and working lives and social avoidance [28]. The results are concerning as the effects of social isolation and disconnection are well known to influence mental health. Together then, this research demonstrates the importance of supporting not only physical, but also social and emotional functioning and support-seeking behaviours in lipoedema.

### Psychological support seeking

Best practice guidelines for lipoedema internationally show psychological support is a key component of lipoedema health management [3, 5, 19] and emotional support and reassurance that lipoedema is not the fault of the individual and referrals to professional psychological support are important [9, 10]. However, only 22% of participants sought mental health support from a psychologist about lipoedema. This could be related to a range of barriers such as stigma in mental health as well as negative experiences with weight stigma in health care, shame, and isolation. Engaging in treatment-seeking behaviour is, therefore, difficult and together with the finding that few GPs provided mental support, this demonstrates that increased awareness and understanding of the psychological effects of lipoedema and its importance to chronic health care is vital.

### Limitations

The international, cross-sectional design provides insight into the self-reported experience of those with lipoedema. It is noted that participants were primarily from the United States and Netherlands, which is to be expected given the strong global presence of the support group networks for lipoedema in these countries. Further, because of the diagnostic ambiguity about lipoedema, it is likely that many low-middle income countries (LMIC) may not have health knowledge of lipoedema and, thus, be able to participate in this research. From this study, the data highlight that broad health knowledge about lipoedema is low and as such is likely to be low and under-represented in LMIC countries. Further, the variables measured were brief and relied on the self-reported presence of mobility issues and mental health concerns such as depression rather than using validated scales or a clinical diagnostic framework. Despite this, the current study showed key areas in which future research may follow up using more sensitive and validated measures to provide further clarity to the clinical interpretation of the findings. It is also noted that data collection took place in 2014–2015 and as such, awareness and recognition by GPs of lipoedema as a valid health condition may have improved due to emerging standards of care and best practice guidelines [2, 5]. As such, increasing awareness and knowledge of lipoedema and how it differs from obesity may increase experiences of appropriate health care support and treatments.

## Conclusions

This study provided a unique description of the lived experience of those with lipoedema and a divergent pattern of experiences was identified between stages. Lipoedema involves physical, emotional, and social constraints that are misunderstood in health care settings. This study profiles symptoms of lipoedema to promote education about the condition and highlight the negative impact of weight bias and stigmatisation to better understand the mental health supports and treatments to promote psychological and social functioning. As such, there is a need to develop targeted interventions aimed at increasing knowledge and awareness of lipoedema and the use of appropriate strategies to promote physical and psychological functioning.
